# Interspecies Comparisons of the Effects of Potential Antiviral 3-Amidinophenylalanine Derivatives on Cytochrome P450 1A2 Isoenzyme

**DOI:** 10.3390/vetsci9040156

**Published:** 2022-03-23

**Authors:** Zsófia Fedor, Anna Szentkirályi-Tóth, Gábor Nagy, Zoltán Szimrók, Eszter Varga, Anna Pászti, Zoltán Pászti, Ákos Jerzsele, Oliver Pilgram, Torsten Steinmetzer, Gábor Mátis, Zsuzsanna Neogrády, Erzsébet Pászti-Gere

**Affiliations:** 1 Department of Pharmacology and Toxicology, University of Veterinary Medicine, 1078 Budapest, Hungary; fedor.zsofia14@gmail.com (Z.F.); anna.szentkiralyi@gmail.com (A.S.-T.); nagy.gabor@univet.hu (G.N.); szimrok.zoltan@univet.hu (Z.S.); eszter.varga06@gmail.com (E.V.); paszti.panni@gmail.com (A.P.); jerzsele.akos@univet.hu (Á.J.); 2 Research Centre for Natural Sciences, Institute of Materials and Environmental Chemistry, 1117 Budapest, Hungary; zpaszti@chemres.hu; 3 Faculty of Pharmacy, Institute of Pharmaceutical Chemistry, Philipps University Marburg, 35037 Marburg, Germany; oliver.pilgram@pharmazie.uni-marburg.de (O.P.); steinmetzer@uni-marburg.de (T.S.); 4 Division of Biochemistry, Department of Physiology and Biochemistry, University of Veterinary Medicine, 1078 Budapest, Hungary; matis.gabor@univet.hu (G.M.); neogrady.zsuzsanna@univet.hu (Z.N.)

**Keywords:** 3-amidinophenylalanine, beagle, cynomolgus monkey microsome, SARS-CoV-2, CYP1A2, hepatocytes

## Abstract

In vitro models of animals vulnerable to SARS-CoV-2 infection can support the characterization of effective antiviral drugs, such as synthetic inhibitors of the transmembrane protease serine 2 (TMPRSS2). Changes in cytochrome P450 (CYP) 1A2 activities in the presence of the potential TMPRSS2/matriptase inhibitors (MI) were measured using fluorometric and luminescent assays. Furthermore, the cytotoxicity of these inhibitors was evaluated using the MTS method. In addition, 60 min-long microsomal stability assays were performed using an UPLC-MS/MS procedure to elucidate depletion rates of the inhibitors. CYP1A2 was influenced significantly by MI-463 and MI-1900 in rat microsomes, by MI-432 and MI-482 in beagle microsomes, and by MI-432, MI-463, MI-482, and MI-1900 in cynomolgus monkey microsomes. The IC50 values in monkey microsomes were 1.30 ± 0.14 µM, 2.4 ± 1.4 µM, 0.21 ± 0.09 µM, and 1.1 ± 0.8 µM for inhibitors MI-432, MI-463, MI-482, and MI-1900, respectively. The depletion rates of the parent compounds were lower than 50%, independently of the investigated animal species. The host cell factor TMPRSS2 is of key importance for the cross-species spread of SARS-CoV-2. Studies of the in vitro biotransformation of TMPRSS2 inhibitors provide additional information for the development of new antiviral drugs.

## 1. Introduction

Recently, it has been discovered that several mammals can be infected with the novel severe acute respiratory syndrome coronavirus-2 (SARS-CoV-2) via angiotensin converting enzyme-2 (ACE2) receptors showing vulnerability mainly to human-to-animal transmission. Animals possessing certain 25 amino acid residues long sequences on the receptor ACE2 similar to those in humans are highly susceptible for SARS-CoV-2 infection, since this sequence is responsible for viral entry into host cells [1,2]. Several species have tested positive for SARS-CoV-2 until now, including dogs, domestic cats, tigers, lions, minks, ferrets, and other animal species, which can be correlated with the human COVID-19 pandemic. It was also confirmed that natural SARS-CoV-2 infection occurred in domestic rabbits with low seroprevalence, based on microsphere immunoassay in addition to the presence of virus replication detected in experimental rabbits, which were only minimally permissive to infection [3,4,5]. Recently, Russia has registered the first vaccine against COVID-19 for animals. Carnivac-Cov is a chemically inactivated vaccine, which confers protection against SARS-CoV-2 infection in animals and, at the same time, reduces the risk for transmission of viral mutation [6,7,8]. In 2021, eight western lowland gorillas were infected in Safari Park, San Diego, USA. A total of nine great apes, including four orangutans and five bonobos, were injected with experimental vaccines developed by veterinary pharmaceutical company Zoetis for the zoo’s emergency use. In Chile lions, tigers, pumas, and an orangutan were vaccinated with an experimental COVID vaccine. Meanwhile, zoos across the United States have started to administer Zoetis vaccines.

Primates, including cynomolgus macaques, have identical amino acids to humans on the receptor ACE2 at their binding site for the virus. As such, they possess a high susceptibility to infection under experimental conditions; however, they were asymptomatic or have shown only mild symptoms, compared with experimentally infected rhesus macaques, cynomolgus macaques, and common marmosets, and it was reported that each one presented a few features of COVID-19 [9]. In addition, other species such as American mink, ferrets, and domestic and large cats also showed high susceptibility, and became infected under natural conditions as well. According to the World Organisation for Animal Health [10], dogs can be infected both naturally (most likely mainly from humans) and experimentally. Occasionally, they can produce clinical signs of infection. In dogs, the infection rate is low, and they cannot transmit SARS-CoV-2 to humans. It is interesting to note that the occurrence of SARS-CoV-2 in rats has not yet been described. 

The One Health approach [11] provides strategies to decrease cross-species transmission of viral infections, including continued surveillance of wildlife and domestic animals, thereby supporting the development of novel vaccines and antiviral drug therapies [12]. SARS-CoV-2 entry is possible via the attachment of a spike (S) protein to host receptor ACE2 and S activation by cleavage via transmembrane protease serine 2 (TMPRSS2) [13,14]. Therefore, one potential anti-SARS-CoV-2 treatment option could be the development of effective TMPRSS2 inhibitors, which would possess a sufficient selectivity against other trypsin-like serine proteases and an acceptable safety profile. Until now, several inhibitor types have been developed for this protease family, including sulfonylated 3-amidinophenylalanine-derivatives [15,16].

Interspecies comparisons of the impact of these protease inhibitors on cytochrome P 450 (CYP) 1A2 in microsomal and hepatocyte models of mammalian species are of crucial importance for further preclinical studies, e.g., for the treatment of SARS-CoV-2 infections, and to interrupt the transmission chain of the virus. In our study, changes in CYP1A2 activities induced by four 3-amidinophenylalanine-derived inhibitors were analysed using microsomes, and two of them (MI-432 and MI-1900) were aplied in hepatocytes of cynomolgus monkeys, beagles, and rats. In addition, depletion rates of the inhibitors were quantitatively determined in microsomal stability assays, which revealed some differences between the species studied.

## 2. Materials and Methods

### 2.1. Preparation of Inhibitor Solutions for Microsomal and Hepatocyte Assays

**Figure 1:**
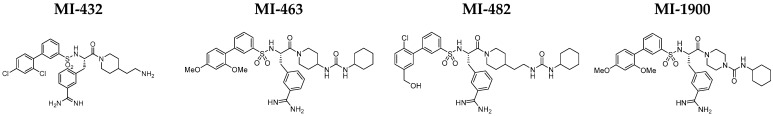
Structures of the 3-amidinophenylalanine-derived matriptase/TMPRSS2 inhibitors.

The inhibitors MI-432, MI-463, MI-482, and MI-1900 were synthesized as described previously for structurally related derivatives, and their chemical structures are presented in Figure 1. Initially, 10 mM stock solutions in dimethylsulfoxide (DMSO) were prepared, and kept at −20 °C. The working solutions of the inhibitors were made freshly from the stock solutions prior to each experiment. After incubation of the microsomes or hepatocytes with the inhibitors at 37 °C in a humidified atmosphere of 5% CO_2_ (using untreated samples as controls), the solutions were subjected to subsequent MTS cytotoxicity, fluorometric or luminescent assays, or to UPLC/MS-MS procedures.

### 2.2. Cytotoxicity Assays in Hepatocytes

Rat, beagle, and cynomolgus monkey cryopreserved primary hepatocytes were obtained from Primacyt Cell Culture Company (Schwerin, Germany). The thawing, plating, and maintenance media were purchased from Lonza Group Ltd. (Biocenter Ltd., Szeged, Hungary). Influence of inhibitors MI-432 and MI-1900 on the viability of primary human hepatocytes was tested. Hepatocytes, grown on a 96-well plate for 24 h, were incubated with the inhibitors at 0, 50, and at 100 µM for 24 h in treated groups. The control cells were incubated only with cell maintenance medium. After removal of the medium and three-fold washing of the cells with phosphate-buffered saline (PBS), 20 μL aliquots of CellTiter 96 AQueous one solution (MTS, Promega, Bioscience, Budapest, Hungary) were pipetted into a 96-well plate, each containing 100 μL of phenol red free medium. The plate was incubated with dye for 2 h. Viability of hepatocytes was measured at 490 nm using an EZ Read Biochrom 400 microplate reader.

### 2.3. CYP1A2 Fluorometric Activity Measurements

The Biovision CYP assay (BioVision, Inc., Kampenhout, Belgium) utilizes a non-fluorescent CYP1A2 substrate capable of transforming into a highly fluorescent metabolite, which can be detected in the visible range. α-naphthoflavone was used as a CYP1A2 reference inhibitor at 6 µM during the experiments. Rat (Sprague–Dawley) and beagle (Gibco, Biocenter, Szeged, Hungary, protein concentration: 20 mg/mL) hepatic microsomal preparations were prepared separately by mixing 25 µL of the obtained rat or beagle microsomal suspension with 2425 µL assay buffer and with 50 µL NADPH generating system (100×). In the case of the cynomolgus monkey microsomal suspension (Primacyt, Schwerin, Germany, protein concentration: 5 mg/mL), the following volumes were applied: in addition to 100 µL of monkey microsomal suspension, the amount of assay buffer and the NADPH generating system were adjusted in order to ensure the original ratios of these components to reach a final volume of 2500 µL. Rat, beagle, and cynomolgus monkey microsomal suspension aliquots (50 µL) were treated with the protease inhibitor 20 µL, 0–1000 µM), the reference compound α-naphthoflavone (20 µL, 30 µM), or the assay buffer without test compounds (20 µL, background control) for 15 min at 37 °C. After incubation, 30 µL of the appropriate CYP1A2 substrate/NADP+ mixture was added to each well, yielding a final reaction volume of 100 µL/well. The protein contents of the obtained microsomes were analysed using bicinchoninic acid protein assay kit (Pierce BCA kit, Thermo Fisher Scientific, Waltham, MA, USA). Tests also confirmed that ≤0.1% DMSO and ≤1% ACN did not cause significant inhibition in CYP1A2. The fluorescence intensities were measured with a fluorometer (Victor X2 2030, Perkin Elmer, Waltham, MA, USA), using λ_Ex_/_Em_ = 406/468 nm. 

### 2.4. CYP1A2 Luminescent Method for Activity Measurements in Hepatocytes

For the measurements of CYP1A2 activities, hepatocytes were grown on 24-well collagen-coated membrane inserts (Costar Transwell-PTFE membrane, 6.5 mm, pore size: 0.4 µm, Merck, Darmstadt, Germany). The hepatocytes were exposed to either 0, 10, 20, and 50 µM MI-432 and MI-1900, or to the known CYP1A2 inhibitor α-naphthoflavone at 10 µM as positive control. The determination of the CYP1A2 activities were performed using the P450-Glo CYP450 Assay (Promega, Madison, WI, USA) according to the manufacturer’s instructions.

### 2.5. UPLC-MS/MS Method for the Determination of the Inhibitor Metabolization

Rat, beagle (Gibco, Biocenter, Szeged, Hungary), and cynomolgus monkey (Primacyt, Schwerin, Germany) liver microsomes were exposed to inhibitors MI-432, MI-463, MI-482, or MI-1900 at 50 µM for 60 min. The final microsomal protein concentration in the suspension was 0.6 mg/mL in each case. The reaction buffer (100 mM phosphate buffer pH 7.4) also contained 1 mM nicotinamide adenine dinucleotide phosphate (NADPH), 5 mM MgCl_2_, 10 mM glucose-6-phosphate (G6P), and 2 IU/mL glucose-6-phosphate dehydrogenase (G6PD). The reaction was terminated by the addition of ice-cold acetonitrile (ACN) with the ratio of sample ACN volume = 1:2. The precipitated protein was removed by centrifugation at 10,000× *g* for 10 min, and the supernatants were used for subsequent analyses. 

The determination of all analytes was carried out using an UPLC/MS/MS system, which consisted of an HPLC apparatus (SCIEX ExionLC^TM^ UPLC, Framigham, MA, USA) and a SCIEX Qtrap 4500 triple-quadrupole mass spectrometer (Framigham, MA, USA) equipped with a SCIEX Turbo V electrospray ionization source. The HPLC columns used were LiChrospher RP Select B (125 × 4.0 mm, 5.0 μm, C8 stationary phase). Next, 0.1% trifluoroacetic acid in ultra-purified water (A) and acetonitrile (B) were used as mobile phases for chromatographic separations applying a gradient elution. The HPLC gradient program was the following: between 0 and 10 min, a ratio of 70% A and 30% B; from 10 to 20 min, linearly increasing to a ratio of 50% A and 50% B; from 20 to 21 min, maintained at a ratio of 50% A and 50% B; from 21 to 22 min, linearly decreasing back to a ratio of 70% A and 30% B; and between 22 min and 30 min, maintained at a ratio of 70% A and 30% B. The run time was 30 min, the column temperature was adjusted to 35 °C, and the flow rate was 1 mL/min.

### 2.6. Statistical Analysis

For statistical evaluation, the R 2.11.1 software package (2010) was applied. Differences between absolute means were evaluated by one-way analysis of variance (one-way ANOVA) with a post-hoc Tukey test, where data were of normal distribution, and the homogeneity of variances was confirmed. Differences were considered significant if the * *p* value was <0.05 marked with * (** *p* < 0.01, *** *p* < 0.001). 

## 3. Results

### 3.1. Effects of the Protease Inhibitors on Monkey Microsomal CYP1A2 Activity 

**Figure 2:**
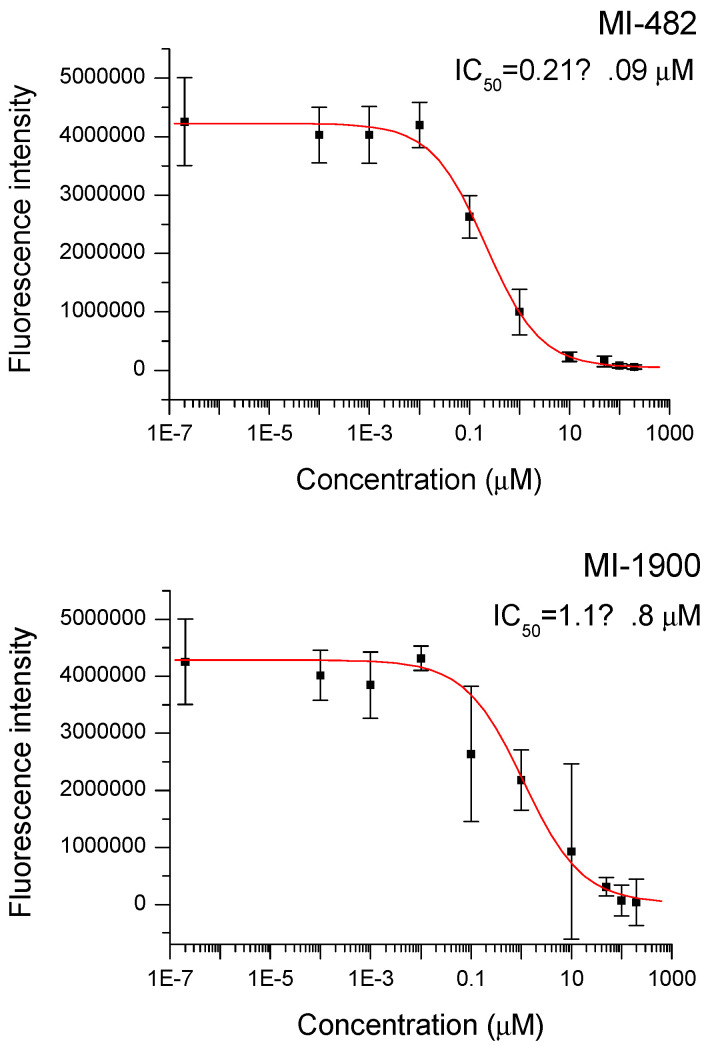
Inhibition of monkey hepatic microsomal CYP1A2 isoenzyme by the utilized protease inhibitors indicated by IC_50_ values. The microsomal preparations were treated with the inhibitors for 15 min at 37 °C. On the Y-axis, the mean fluorescence intensity values ± SD (*n* = 3) are shown.

The 3-amidinophenylalanine-derived protease inhibitors were added to monkey microsomal preparations for 15 min. It was observed that CYP1A2 isoenzyme activities in monkey microsomes exposed to all four inhibitors were significantly influenced. The IC_50_ values were found to be 1.30 ± 0.14 µM, 2.4 ± 1.4 µM, 0.21 ± 0.09 µM, and 1.1 ± 0.8 µM for MI-432, MI-463, MI-482, and MI-1900, respectively, showing a significant CYP1A2 isoenzyme inhibition. Figure 2 describes the concentration-dependent changes in fluorescence intensities, as well as the determination of the IC_50_ values for all four inhibitors. 

### 3.2. Influence on Rat and Beagle CYP1A2 Activities

**Figure 3:**
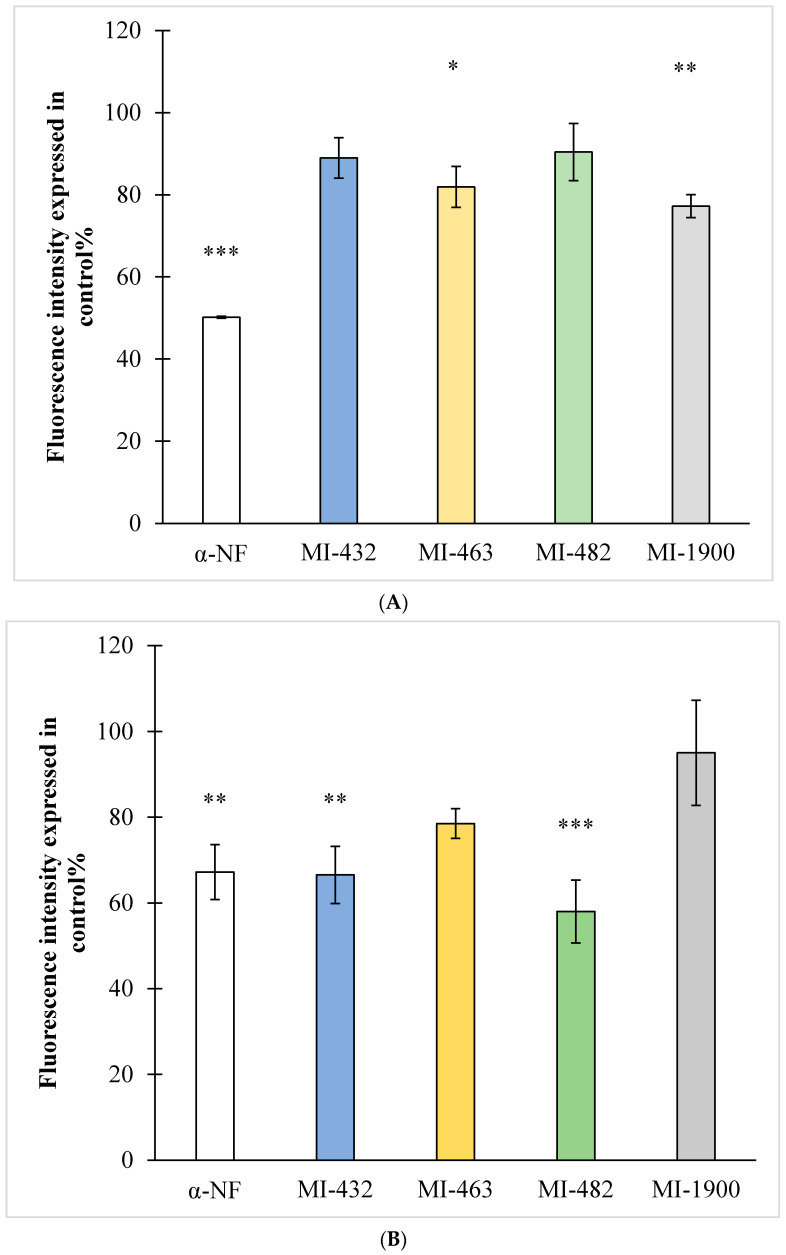
The effect of the protease inhibitors at 50 µM on rat (**A**) and beagle (**B**) hepatic microsomal CYP1A2 isoenzyme activity. The microsomal preparations were treated by the inhibitors for 15 min at 37 °C. The reference inhibitor α-naphthoflavone (α-NF) was used at a concentration of 6 µM, and significantly suppressed CYP1A2 activities in rat (*** *p* < 0.001) and beagle microsomes (** *p* = 0.002), (* *p* < 0.05). The shown data are the mean fluorescence intensities expressed as a percentage of the untreated control ± SD (*n* = 3).

Inhibitors at 50 µM were added to rat and beagle microsomal preparations for 15 min, using α-naphthoflavone as a CYP1A2 reference inhibitor at 6 µM (positive control). It was observed that rat CYP1A2 isoenzyme activities were suppressed by the administration of inhibitors MI-463 (*p* = 0.025) and MI-1900 (*p* = 0.005); however, MI-432 and MI-482 did not inhibit CYP1A2 function (*p* > 0.05 in each case) (Figure 3A). When using beagle microsomal preparations, the inhibitors MI-432 and MI-482 reduced the activity of CYP1A2 (*p* = 0.003 and *p* < 0.001, respectively), whereas the other two inhibitors did not exert any significant effect (Figure 3B). 

### 3.3. Depletion of the Protease Inhibitors in Microsomal Stability Assays

**Figure 4:**
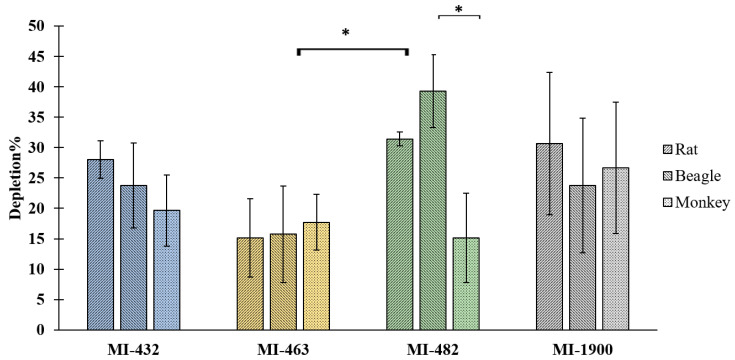
Interspecies comparison of inhibitor depletion in microsomes. The inhibitors at a concentration of 50 µM were incubated for 60 min with the microsomal preparations. The average degradation rate of inhibitor MI-482 significantly differed in beagle and monkey microsomes (* *p* = 0.025). Significant differences were found in depletion percentages of compounds MI-463 and MI-482 in beagle microsomal samples (* *p* = 0.032). The measured data are represented as mean of the inhibitor depletion in % ± SD (*n* = 3).

Based on the 60 min microsomal stability data, it was ascertained that all four protease inhibitors were metabolized during exposure to hepatic microsomal CYP isoenzymes. The depletion percentage values of the compounds were found to be 28 ± 3.1%, 15.1 ± 6.5%, 31.4 ± 1.1%, and 30.7 ± 11.7% in rat microsomes, 23.8 ± 7%, 15.7 ± 8%, 39.3 ± 6%, and 23.7 ± 11.1% in beagle samples, and 19.6 ± 5.8%, 17.7 ± 4.6%, 15.1 ± 7.3%, and 26.7 ± 10.8% in monkey microsomes for inhibitors MI-432, MI-463, MI-482, and MI-1900, respectively. No interspecies differences were observed in the CYP1A2 activities of rat, beagle, and monkey microsomes exposed to MI-432, MI-463, and MI-1900. Inhibitor MI-482 was metabolized faster in beagle microsomes compared to conversion rates in monkey microsomes (*p* = 0.025). Comparing the percentage of depletion in the beagle microsomal assay, the highest transformation rate was detected in case of MI-482, which significantly differed from that of MI-463 (*p* = 0.032). (Figure 4). 

### 3.4. Assessment of Cell Viability

**Figure 5:**
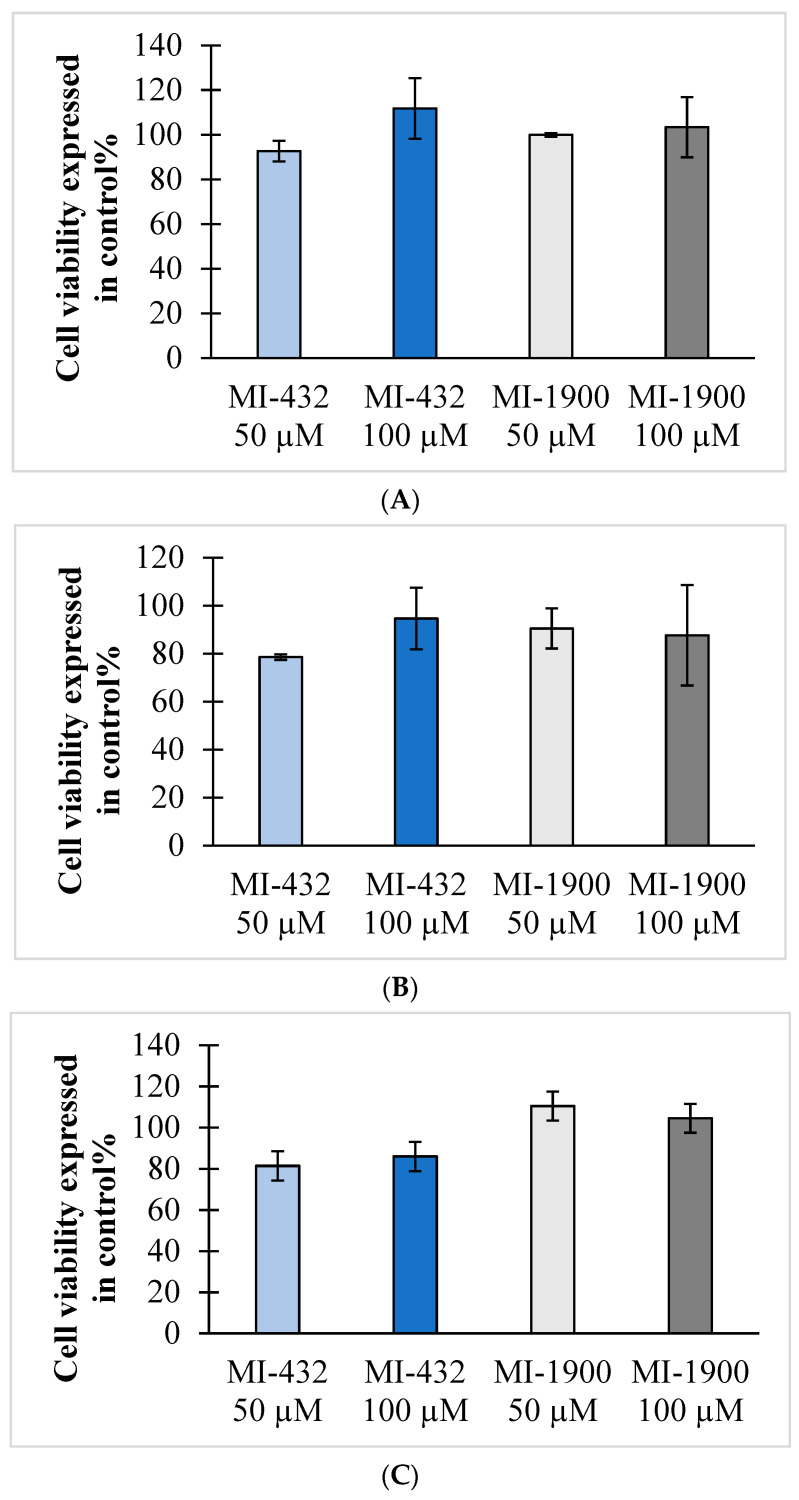
Cytotoxicity test of the 3-amidinophenylalanine-derived protease inhibitors on hepatocytes of different species. The hepatocytes of rat (**A**), beagle (**B**), and cynomolgus monkey (**C**) were incubated for 24 h in the absence (control, set to 100%) or in the presence of inhibitors MI-432 and MI-1900 at concentrations of 50 μM and at 100 μM. Data represent the average cell viability values expressed as a percentage of the control ± SD. No significant differences were found between the control experiments and inhibitor-exposed hepatocytes (*n* = 3).

Cell cytotoxicity assays were performed to evaluate if a 24 h long administration of the inhibitors up to 100 µM concentration could cause significant cell death in hepatocytes of mammalian species. It was found that the selected inhibitors (MI-432 and MI-1900) can safely be used at a concentration of 50 µM for 24 h in rat (Figure 5A), beagle (Figure 5B), and cynomolgus monkey hepatocytes (Figure 5C); thus, this concentration could be applied in further experiments to characterize the effects of matriptase/TMPRSS2 inhibition using in vitro hepatic models of different animals.

### 3.5. Evaluation of CYP1A2 Changes in Hepatocytes Treated with Inhibitors MI-432 and MI-1900

**Figure 6:**
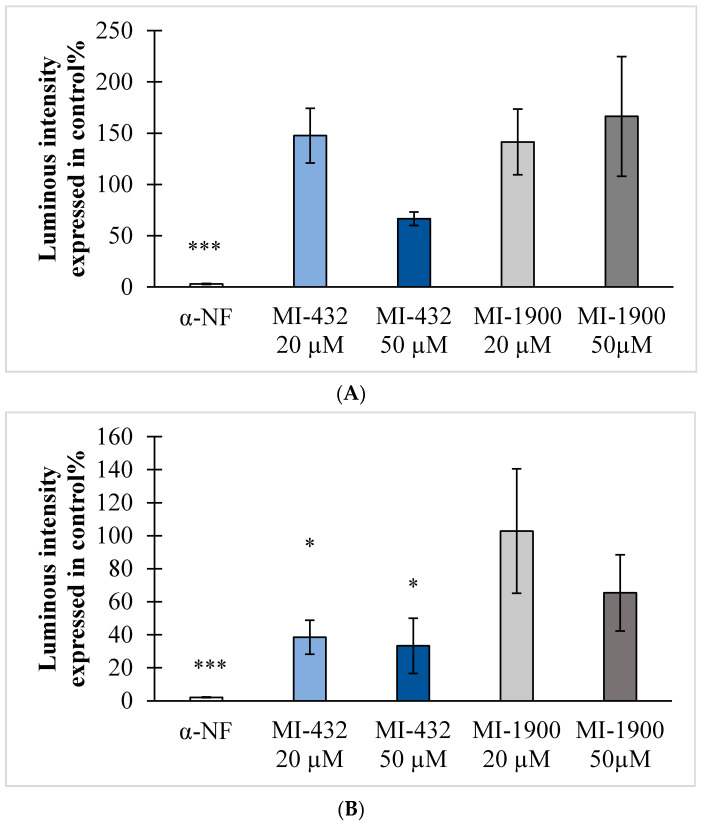

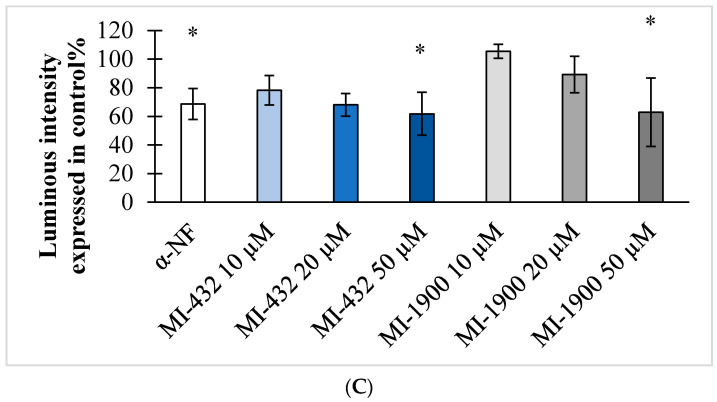
Determination of luminous intensities from CYP1A2 activities in hepatocytes of different animals. (**A**) In rat hepatocytes, no significant differences were found in luminous intensities between the inhibitor-treated and control groups (*p* > 0.05). (**B**) In beagle hepatocytes exposed to inhibitor MI-432 at concentrations of 20 and 50 µM, a significant suppression of CYP1A2 was found (* *p* = 0.047 and * *p* = 0.027, respectively). (**C**) In cynomolgus monkey hepatocytes, the inhibitors MI-432 and MI-1900 reduced CYP1A2 activities at a concentration of 50 µM (* *p* = 0.032 and * *p* = 0.039, respectively). The positive control α-naphthoflavone (α-NF) at 10 µM could decrease CYP1A2 activity significantly in rat (*** *p* < 0.001), beagle (*** *p* < 0.001), and cynomolgus monkey (* *p* = 0.028) hepatocytes. The data are shown as the mean luminous intensity values ± SD (*n* = 3).

Hepatocytes were exposed to inhibitors MI-432 and MI-1900 with proven in vitro antiviral effects for 2 h at different concentrations (0, 20, and 50 µM in rat and beagle hepatocytes, and at 0, 10, 20, and 50 µM in cynomolgus monkey hepatocytes). It was found that inhibitors MI-432 and MI-1900 affected CYP1A2 function dependent on species. Based on chemiluminescent data, the activity of CYP1A2 did not change after inhibitor treatment in rat hepatocytes (*p* > 0.05 in each case, Figure 6A). It was also confirmed that the inhibitor MI-432 at concentrations of 20 µM and 50 µM significantly inhibited CYP1A2 function (*p* = 0.047 at 20 µM and *p* = 0.027 at 50 µM) in beagle hepatocytes (Figure 6B). In cynomolgus monkey hepatocytes, each applied inhibitor could significantly decrease the activity of CYP1A2 at 50 µM (*p* = 0.032 for MI-432 and *p* = 0.039 for MI-1900, Figure 6C), compared to control values. As a positive control, the selective CYP1A2 inhibitor α-naphthoflavone at 10 µM was used, and could significantly suppress CYP1A2 activities in each type of hepatocyte (*p* < 0.001 in rat and in beagle hepatocytes, and *p* = 0.028 in cynomolgus monkey hepatocytes).

## 4. Discussion

The COVID-19 pandemic has led to a global public health concern. Successful management of zoonotic infectious diseases can save both human and animal lives. 

The One Health approach, which includes collaborative application of human and veterinary pharmacological and clinical knowledge, can provide strategies against zoonotic coronaviruses. Several already-authorized drugs were rapidly repurposed and tested against SARS-CoV-2-mediated human infection; however, most of these drugs showed limited efficacy. It was previously proven that proteolytic cleavage of the influenza virus hemagglutinin (HA) and the S of SARS-CoV or MERS-CoV by cellular serine proteases, such as furin and/or TMPRSS2, could be addressed for the treatment of influenza, SARS-CoV, and Middle East respiratory syndrome coronavirus (MERS-CoV) infections [17,18,19,20]. The S protein of SARS-CoV-2 is proteolytically processed into functional fragments, and TMPRSS2 activates the S protein at the S2′ site, thus enabling the fusion of viral and cellular membranes, as well as virus entry into the host cells [13,21,22]. SARS-CoV-2 titers were reported to be reduced in infected human airway epithelial Calu 3 cells in a dose-dependent manner when exposed to the 3-amidinophenylalanine-derived inhibitors MI-432 and MI-1900. Decrease in SARS-CoV-2 titers was most likely via suppression of TMPRSS2 [21], suggesting that inhibition of this trypsin-like serine protease could be an effective strategy against SARS-CoV-2 infections. In this research work, primary hepatocytes of different animal species were exposed to these two antiviral MI compounds, and the absence of their cytotoxic effects was confirmed at up to 100 µM in vitro. In our previous studies using intestinal epithelial cells (HIEC and IPEC-J2) and primary human hepatocytes (PHH), it was proven that administration of two 3-amidinophenylalanine analogues, such as inhibitors MI-1900 and MI-1907, up to a concentration 50 μM did not cause significant cell death, and also did not result in elevation in extracellular hydrogen peroxide levels in intestinal epithelial cells [23,24]. Similarly, redox homeostasis of a chicken hepatic cell model exposed to MI-460 and MI-432 was only transiently disturbed, and then was almost completely compensated after 24 h [25]. 

The One Health concept applies multidisciplinary knowledge to support animal and human health care efforts in order to prevent the further occurrence of the COVID-19 pandemic; however, officially no animal reservoir has been reported yet. In a longer time frame, the development of anti-SARS-CoV-2 drugs is required for the successful treatment of human patients, as well as endangered wild species and companion animals. Therefore, the development of appropriate animal models for COVID-19 were needed in order to support the testing of vaccines and therapeutic agents. Mouse ACE2 cannot successfully bind the viral S of SARS-CoV-2, thus mouse models can be used only when modifications are introduced, such as the expression of human ACE2 in genetically modified mice or the alteration of the S of SARS-CoV-2. In ferrets and Syrian hamsters, virus replication was detected in the upper respiratory tract at very early stage, and could be observed for two weeks [26,27,28]. Frequently used non-human primate models for COVID-19 include rhesus macaques, cynomolgus macaques, and African green monkeys [29,30]. In these species, viral replication was detected in both the upper and lower respiratory tract, accompanied by pneumonia and mild clinical disease. It has been recently reported that the COVID-19 cynomolgus monkey model could also reflect the age-related differences of infection in human [31]. It was also found that mink, cats, and dogs were susceptible to infection with SARS-CoV-2; thus, models derived from these species provide opportunities for effective screening of both vaccines and drug candidates. Dogs and humans have similar ACE2 receptors, therefore dogs might be a potential intermediate host. However, the occurrence rate of COVID-19 in dogs was very low, and SARS-CoV-2-mediated infection in this species appeared to be asymptomatic [2,32,33]. Pigs, chickens, and ducks did not appear to show COVID-19 pathophysiology due to a lack of virus replication and spread [34]. Preclinical investigations prior to clinical trials are required for the characterization of potential antiviral drugs, including both in vitro studies as well as in vivo methods with rodents and dogs.

In our study rat, beagle, and cynomolgus monkey hepatic microsomes were used to determine the impact of 3-amidinophenylalanine-derived inhibitors of trypsin-like serine proteases such as TMPRSS2 on CYP1A2. The cynomolgus macaque is one of the closest animals to humans, considering the pathophysiological features of COVID-19, rendering it a model animal for SARS-CoV-2 biomedical research [9,35,36]. In our work, it was observed that CYP1A2 in the cynomolgus monkey microsomal model was inhibited by the administration of inhibitors MI-432, MI-463, MI-482, and MI-1900 at a concentration of 50 µM. All determined IC_50_ values were lower than 5 µM in each case. In accordance, CYP1A2 activity was suppressed significantly in cynomolgus monkey hepatocytes exposed to inhibitors MI-432 and MI-1900 at a concentration of 50 µM. 

It is well documented that the expression patterns of CYP isoenzymes are highly dependent on species [37,38]. The changes in the activity of CYP1A2 were traced after the application of MI compounds in our study, as this isoenzyme was proven to be present in rats, monkeys, and dogs, similar to humans [38]. CYP2C subfamily members CYP2C9, CYP2C19, and CYP3A4 can be found in humans, and only their orthologous forms are present in rats, dogs, and monkeys [39,40,41]. 

In our work, rat and beagle hepatic microsomal preparations were treated with 3-amidinophenylalanine-derived inhibitors, and it was observed that rat CYP1A2 was significantly inhibited by inhibitors MI-463 and MI-1900, and beagle CYP1A2 was blocked by MI-432 and MI-482, indicating interspecies differences in the CYP1A2 modulatory effects by these compounds. Notably, compound MI-432 inhibited the activity of the CYP1A2 isoenzyme both in beagle microsomal preparations and in beagle hepatocytes, similar to the inhibitory effect of all four compounds found in in vitro models of cynomolgus monkeys. Interestingly, rat microsomes and hepatocytes responded differently to the administration of inhibitors MI-432 and MI-1900, as microsomal CYP1A2 activity was only significantly suppressed by MI-1900. In contrast, this inhibitory effect was not detected in hepatocytes. Based on in vitro findings of microsomal stability data, it was ascertained that each investigated protease inhibitor was significantly metabolized by hepatic microsomes, and the strongest depletion rate was found for analogue MI-482 in beagle microsomal preparations. 

## 5. Conclusions

In our work, the involvement of 3-amidinophenylalanine-derived inhibitors in modulating the CYP1A2 isoenzyme activity was elucidated. To our knowledge, this is the first study of rat, beagle, and cynomolgus monkey hepatic microsomes and hepatocytes focusing on the effects of sulfonylated 3-amidinophenylalanine-derived inhibitors on CYP1A2-mediated biotransformation pathways. The strongest effect of the protease inhibitors on CYP1A2 activity was observed in cynomolgus monkey microsomes, since each of the four inhibitors has got relatively potent IC_50_ value <5 µM. The depletion rates of the applied inhibitors remained <50% in each case by the end of a 60 min-long microsomal stability assay. According to hepatocyte-based cytotoxicity data, the inhibitors MI-432 and MI-1900 could be safely applicable in vitro in a particular concentration range, which appeared to be effective against SARS-CoV-2 in Calu-3 cells. It is interesting to note that the tested 3-amidinophenylalanine-derived protease inhibitors could influence the CYP1A2 activities of the studied species to a different extent in spite of their similar chemical structure. In future, a further development and comprehensive preclinical characterization of these potential TMPRSS2 inhibitors, including investigations of their influence on various CYP isoenzyme activities, are needed to identify effective antiviral drugs against SARS-CoV-2. The most promising compounds should be tested in suitable in vivo models of mammalian species which are prone to SARS-CoV-2 infections.

## Data Availability

Data are available from the corresponding author upon reasonable request.
